# Reminiscences of the Insane

**Published:** 1886-11-20

**Authors:** 


### Reminiscences of the Insane.

By a Mental Physician.

Conversations and Letters.

Of the various autobiographical narratives and the letters of insane patients which have passed into our hands, very few are suitable for publication. We may, however, extract from them some interesting and valuable passages, which may, perhaps, be suited to the columns of The Hospital. A patient will often write what he does not speak. He reveals his delusions involuntarily, while in conversation he may succeed in concealing them. On the other hand, a patient's letter sometimes astonishes one by the easy flow of ideas, the natural tone, and the entire avoidance of the morbid notions for which he had been placed under care. We have known a lady who in conversation answered the simplest questions incorrectly—and indeed obviously laboured under dementia—write an absolutely coherent and intelligent letter to her brother at college; such an epistle, in fact as would have led us, if ignorant of the case, to assert in a court of law that the writer appeared to be perfectly *compos mentis.*

Verbal Proliferation.

Some of the compositions of patients are marked by an absurd multiplication of words, a verbal proliferation which may be viewed as a satire on too many authors not having the same excuse. Thus an insane gentleman in an asylum in which we were resident, wrote, "Whom Nature commends to the assistance and favour of others, in general she commends by means of a beauty, cleverness, dexterity, or handsomeness in the appearance of the thus more attractive-made subject. This may be the cause that most, if not all, women and perhaps some men, display as they do such self-disesteem and disgust, that some, having set their happiness on a fancy in which they are disappointed, show these feelings to an excess that produces in them a disposition malevolent to or towards others, and that I unknowing, am known. Whether or not this or any of its above-mentioned effects, or the generally, if not to me all-powerful 'number one' feature of man's nature, or all these combined, made me so bad as I was, I hesitate not to decide in my own mind; and now, with truth and justice to my feelings, both write and say on the subject that nobody was or can be worse than I especially was or am, unless she, he, or it was or can be worse than I especially was or am. I doubt not that it was, and has always by everybody been thought, that I neither had nor have the least particle of truth in any of my ideas. I, however, both write and speak, and wrote and spoke, what were and are my feelings as well as I could and can at the time." The same patient wrote, "How much soever individuals of the human race may be against me, the *race* is not against, but for me. Whatever evil happened to me, happened from some cause of individual contact, or more properly opposition." Again, "I live in a world that can neither pity nor spare; therefore I am beyond everybody's comprehension. I am totally unconnected with any other creature's interest or interests."

Features of Insane Composition.

The ordinary reader can hardly fail to detect a touch of incoherent thought in the first paragraph quoted; and in fact the writer was partially demented at the time. The whole paragraph is mere verbiage, and ends in nothing: and this is precisely what characterises the great mass of insane composition. But, on the other hand, the succeeding paragraphs, written during the same mental state, are consistent with the possession of considerable mind, although betraying delusions of a kind very common in the insane. In this instance, the weakness of mind which was actually present, did not allow of the melancholy tone of these latter paragraphs being more than superficial. Happily, not a little of the expressed misery of the insane is automatic, and may be but the dying echo of a once acutely-felt sorrow. I note one more memorandum of the same patient, which is worth transcribing: "I am already," he writes, "what I was born to be, viz., considered, minded, regarded, or thought of, in one way or another; and that being the only ground of satisfaction I can ever possibly have, I may, if I please, take no other."

The Sublime and Ridiculous.

One day we observed this patient in the ward extremely amused at something, and asked him what it was. He replied, "I was only thinking of something very droll." Well, but what? "I was thinking about what I often think about—the origin of evil." How?—the cause of it? "Yes; how it is things are not all right, and why there should be any evil." Well, why is it? "Why, I sometimes think it must be because people don't take pains with their writing; if everyone wrote well, I don't think there would be any evil." An excellent illustration this of the combination of the sublime and the ridiculous which is so often met with among the insane. A striking observation is made, and one awaits the hatching of a brilliant thought; but alas! too often, there is the same grotesque descent from a philosophical eminence as in the example we have just given. Great wits may sometimes be near allied to madness, but it cannot honestly be said that from states of madness proceed great wit, in Dryden's sense of the word. "It is impossible for the mind to conceive of a mad Shakespeare," said Charles Lamb. However, we are inclined to think that a great many who have written on the origin of evil have not talked less nonsense than the partially demented patient in ——— Asylum. And, assuredly, bad writers are an evil to all who have anything to do with them, more especially printers, on whom they inflict loss of temper, loss of time, and loss of eye-sight. So our friend was not so very far wrong after all, when one comes to think of it.

*(To be continued.)*

---

A Combination Dose.—Mark Twain says that one of his men happening to fall sick, he went to his medicine-chest, where he found, from the printed instructions, that his patient should be given a tablespoonful of No. 15. He found that No. 15 bottle in the chest was empty, but as 8 and 7 were nearly full, he took these, mixing half a dose of each. Strange to say, that, though 8 and 7 make 15, Mark Twain's patient died.

